# Control of Cowpea Weevil, *Callosobruchus Maculatus* (F.) (Coleoptera: Bruchidae), Using Natural Plant Products

**DOI:** 10.3390/insects6010077

**Published:** 2014-12-31

**Authors:** Bamphitlhi Tiroesele, Kesegofetse Thomas, Seipati Seketeme

**Affiliations:** 1 Department of Crop Science and Production, Botswana College of Agriculture, Private Bag 0027, Gaborone, Botswana; E-Mail: kesetommy@gmail.com; 2 Ministry of Agriculture, Department of Agricultural Research, Private Bag 0033, Gaborone, Botswana; E-Mail: feli67032@gmail.com

**Keywords:** cowpeas, infestation, management, weevils

## Abstract

A laboratory study was conducted to investigate the effects of natural products on the reproduction and damage of *Callosobruchus maculatus,* the cowpea weevil, on cowpea seeds at Botswana College of Agriculture in Gaborone, Botswana. The cowpea variety Blackeye was used in the study. Fifty grams of each plant product (garlic, peppermint and chilies) was added to 500 g of the cowpea seeds. Findings of this experiment revealed that chilies and garlic had negative effects on cowpea weevils for all parameters measured. Peppermint also showed significant reduction in the F_1_ progeny of the cowpea weevils but with less effect on weevils than garlic and chilies. The results indicate that these plant products have the potential to protect cowpea seeds from cowpea weevils’ damage compared to when the seeds are left or stored unprotected. They should, therefore, be included in pest management strategies for cowpea weevil in grains stored on-farm in rural tropical and subtropical regions.

## 1. Introduction

Cowpea, (*Vigna unguiculata* (L) Walpers, Fabaceae), is an important edible legume crop in many parts of the world especially in tropical and subtropical regions. It is used as human food due to its high protein content [1] and also as livestock feed to make silage and hay. Cowpea is the third most important crop in Botswana after maize, *Zea mays* L., and grain sorghum, *Sorghum bicolor* (L.) Moench (both Poaceae) [2]. Production of cowpea in Botswana is about 300–355 kg/ha. It is an annual herbaceous legume with trifoliate leaves. It is a drought tolerant and short warm weather crop well adapted to the drier regions where other food legumes do not perform well [3]. It requires annual rainfall of about 750–1100 mm [4].

Cowpea production is affected by insect pests and disease infestations which lead to economic losses. Insect damage is the major constraint to cowpea grain production in most cowpea producing nations [5]. The major insect pests that can cause economic loss are cowpea aphids *(Aphis craccivora)*, leafhoppers *(Empoasca spp)*, thrips (*Megalurothrips sjostedti—*Synonym: *Taeniothrips sjostedti*), flower eating beetles *(Mylabris spp. and Coryna spp.*), blister beetles (*Hycleu slugens*), green stink bugs (*Nezara viridula*) and cowpea weevil* (Callosobruchus maculatus)*. The cowpea weevil, *Callosobruchus maculatus* (F.) (Coleoptera: Bruchidae), is a cosmopolitan field-to-store pest ranked as the principal post-harvest pest of cowpea in the tropics [6]. It causes substantial quantitative and qualitative losses manifested by seed perforation and reductions in weight, market value and germination ability of seeds [7].

In order to reduce serious losses experienced during storage, various techniques and control methods have been developed and more are still being developed. Management of cowpea seed storage pests relies heavily on the use of chemical insecticides. However, most of the small scale farmers have not adopted these new techniques due to some financial and technical reasons. Insecticides also have negative impact on the environment, humans and non-target organisms. Therefore, there is a need to develop cheap, safe and easy methods of protecting stored cowpeas against cowpea weevil.

Resource-poor farmers in Africa employ a range of traditional methods such as use of ash, sand, dry pepper and botanical extracts. Naturally occurring plant products have been used to protect agricultural products against pests for many years in some parts of the world; many authors have reported insecticidal effects of plant products against a broad range of pests. Some of the techniques that can be explored include the use of plant products such as garlic, peppermint and chilies. Aromatic plants have both medicinal and aromatic properties and contain a variety of volatile oils which have insecticidal, anti-feedant and repellent effects on insect pests. The chemical repellency hypothesis states that non-host plant odors repel herbivores by disrupting their ability to locate or feed on the host plant [8]. The plant products used for this study produce odors that are believed to repel weevils, thereby preventing them from attacking cowpea seeds.

There is limited information on the use of the plant products as an alternative control method for controlling weevils in storage. The use of plant products may offer a sustainable, environmentally friendly and safer alternative to synthetic insecticides. This experiment was aimed at determining the effects of several plant products on cowpea weevil reproduction. This study will also create awareness of the value of plant products as a control method for cowpea weevils in smallholder farmers’ storage facilities.

## 2. Experimental

### 2.1. The Experimental Site

The experiment was carried out in the entomology laboratory at Botswana College of Agriculture using reared population of *C. maculatus.*

### 2.2. The Insects

Adult cowpea weevils were collected from the Seed Multiplication Unit in Sebele from infested Tswana cowpea variety. The stock culture of *C. maculatus* raised by placing 100 unsexed adults in two-liter jars half full of disinfected Tswana cowpea seeds. Muslin cloth was used to cover the top of the jars so that cowpea weevils could not escape. These parent cowpea weevils were allowed to mate for seven days under laboratory conditions (25–28 °C and 60%–90% relative humidity) and lay eggs, after which they were removed. A day after emerging, the insects were sexed by the examination of the elytral pattern [9]; females are maculated with four elytral spots whereas males are plain with less distinct spots.

### 2.3. Cowpea Seeds

The cowpea seeds were bought at Spar superstore at Airport Junction mall (Gaborone) and the seeds were checked to ensure that they were not infested by visual observation for presence of eggs or any suspicious material.

### 2.4. Plant Products

The three plant products, that is, garlic, chilies and peppermint which were used as treatments for this study were bought from Spar and Checkers Superstores at Airport Junction mall (Gaborone) and were kept in a refrigerator. Each plant product was cut into small pieces using a blade and a chopping board. Garlic and chilies piece weighed approximately 0.55 g and 025 g each, respectively. Peppermint leaves were also sliced to small pieces weighing approximatelly 0.035 g each.

### 2.5. Experimental Test

Fifty grams of each treatment plant material prepared was added to 500 g of cowpea seeds in a small glass bottle. The cowpeas were shaken thoroughly to ensure uniform treatment plant material coverage of seeds. Eight replications of 50 g of treated cowpea seeds were placed in bottles with a lace top cover for each plant product. Eight replications without any plant treatment material were used as the control. Five pairs of newly emerged adults were then introduced in each container of cowpea.

### 2.6. Data Collection

The total number of eggs laid was counted daily for four days when generally there were no more adults alive. The numbers of both hatched and un-hatched eggs were also counted under the microscope. Number of damaged seeds was counted looking at the exit holes found on 15 randomly selected seeds of all replications. The whole seeds per replicate were weighed after the experiment and this weight was subtracted from the original weight to find the weight loss of cowpea seeds.

### 2.7. Data Analysis

Data were analyzed using mixed model procedures (PROC MIXED in SAS [10]). Multiple comparisons were done on least square means. All the comparisons was considered significant when *p* ≤ 0.05.

## 3. Results

The plant products significantly reduced the number of eggs laid by cowpea weevils on the seeds (*F*_3,28_ = 145.96, *p* = 0.0001). Fewest eggs were laid on the chilies treatment, but the number of eggs laid on the garlic treatment was not significantly different (Table 1). Significantly more eggs were laid on the peppermint treatment, and still more were laid on the control. The percentage of eggs hatching followed a similar trend, with lowest egg hatch on the chilies treatment, followed by garlic, peppermint, and the control (Table 1).

**Table 1:** Effects of natural plant products on the number of eggs laid by the cowpea weevil and percentage of eggs hatched.

Treatments	Number of Eggs Laid	% Eggs Hatched
Control	219 ± 4.15 ^a^	91.86 ± 3.37^ a^
Garlic	119 ± 2.85 ^c^	84.19 ± 2.45^ b^
Chilies	107 ± 1.98 ^c^	80.83 ± 6.47^ b^
Peppermint	153 ± 3.25 ^b^	87.36 ± 5.07^ a^

* Means ± SE within the column followed by the same small letter are not significantly different (*p* ≤ 0.05).

As expected, F_1_ progeny emergence was significantly reduced by the plant product treatments (*F*_3,28_ = 265.93, *p* < 0.0001). Following the trend seen with oviposition, the fewest progeny were produced on the chili and garlic treatments, both of which were significantly lower than the peppermint treatment, which in turn was lower than the control (Table 2). The mortality of the progeny followed a similar trend, with higher mortality observed on all treatments compared with the control. The highest mortality was observed on garlic and chilies (Table 2). A significant reduction in the exit holes was exhibited on the garlic and chilies treatments compared with the control and peppermint treatments (*F*_3,28_ = 265.93, *p* = 0.0001) (Table 2). The highest number of exit holes was observed on the control but peppermint treatment was not significantly different from it.

Exposure of the seeds attacked by weevils to the plant products treatments significantly reduced the seed weight (*F*_3,28_ = 265.93, *p* = 0049). The lowest weight loss was observed on garlic and this was not significantly different from the weight loss of chilies and peppermint treatments. Significantly high weight loss was shown on the control, but this was not significantly different to the peppermint treatment (Table 3).

**Table 2:** Effects of natural plant products on progeny production, weevil mortality and number of exit holes on cowpea seeds.

Treatment	Progeny Production	Percent Weevil Mortality	Number of Exit Holes on the 15 Selected Seeds
Control	1116 ± 4.15 ^ a^	19.62 ± 6.63^ a^	35 ± 3.37 ^a^
Garlic	856 ± 3.76 ^ c^	87.50 ± 5.14^ c^	17 ± 2.57 ^b^
Chilies	770 ± 4.15 ^ c^	84.94 ± 2.24^ c^	9 ± 3.15 ^b^
Peppermint	822 ± 2.07 ^ b^	59.25 ± 5.62^ b^	29 ± 2.87 ^a^

* Means ± SE within the column followed by the same small letter are not significantly different (*p* ≤ 0.05).

**Table 3:** Average weight loss of cowpea seeds treated with natural plant products and exposed to attack by the cowpea weevil, *C. maculatus*.

Treatment	Cowpea Seeds Average Weight Loss (Grams)
Control	10 ± 1.11 ^a^
Garlic	6 ± 0.92 ^b^
Chilies	4 ± 1.20 ^b^
Peppermint	7 ± 1.00 ^a,b^

* Means ± SE within the column followed by the same small letter are not significantly different (*p* ≤ 0.05).

## 4. Discussion

The results of this experiment showed that treatments differed in number of eggs laid, the percent mortality of eggs hatched, total number of cowpea weevils found on seeds, percent mortality of cowpea weevils, number of exit holes on seeds and weight loss of cowpeas. It has been shown in this experiment that chilies and garlic had similar detrimental effect on cowpea weevils for the parameters measured. This, therefore, implied that these plant products negatively impacted the weevils more than peppermint and control. It can, therefore, be mentioned that chilies and garlic can better protect cowpea seeds from cowpea weevil damage than if seeds are left or stored unprotected. Even though peppermint had less negative effect on the weevils than garlic and chilies, it also showed significant reduction in number of eggs laid, percent eggs hatched, total number of cowpea weevils found on seeds, number of exit holes on seeds, weight loss of cowpeas and increased percent mortality of cowpea weevils compared to untreated seeds. In some parameters such as number of exit holes on seeds and weight loss of cowpea seeds, peppermint did not show any effect on cowpea weevils.

The reduction in the egg laying by the plant products (garlic and chilies) might be due to toxicity of the plant materials to the weevils rather than deterrence of oviposition. This is because, as it can be seen from Table 2, these plant materials resulted in less progeny produced and caused highest mortality which can be linked to toxicity of the plant materials. It should be noted that even though chilies and garlic showed a stronger effect on cowpea weevils when applied on the seeds, they did not eliminate the cowpea weevil’s presence or occurrence of their effect on the seeds but rather reduced weevils numbers and their effect on the seeds to significantly low levels as compared to unprotected seeds. The implication of these effects is that garlic and chilies as well as other plant materials used as treatment in this experiment do not offer total exclusion of cowpea weevil but rather they lowered the weevil numbers and effects on the cowpea seeds.

The reduction in the F_1_ progeny exhibited by garlic and chilies implies that there will be reduction in the damage caused by the weevils on the seeds hence offer a better storage treatment than when left untreated. Garlic and chilies are common plants grown by locals in their backyards, so they are readily available seed treatment sources. In addition, garlic, chilies and peppermint have shown little reduction in percent egg hatch compared to the untreated seeds. This means that they do not have much detrimental effect on the egg hatchability. This will thus result in hatching of a progeny that can further affect seeds in storage.

The findings in this experiment are similar to those of Cowles* et al.* [11] who revealed that powdered chili pepper deterred the onion fly from laying eggs on the base stem of onion. Weissenberg* et al.* [12] stated that chilies contain the compound capsaicin, which reduced the growth of the spiny bollworm, *Earias insulana.* Oleoresin from capsicum has been reported to be effective as a repellent against cotton pests [13]. Capsaicin has also been reported to kill insects by causing membrane damage and metabolic disruption and to affect the nervous system of invertebrates [14]. In addition, Huang* et al.* [15] reported that garlic contains the compounds methylallyl disulfide and diallyl trisulfide. They stated that these compounds reduced egg hatching of *T. castaneum* and the subsequent emergence of progeny. Khani* et al.* [16] reported that the major compounds found in peppermint were menthol, isomenthone, limonene and cineole. Monoterpenoids have strong toxicity to insects due to high volatility and lipophilic properties which make them penetrate into insects rapidly and interfere with physiological functions. Limonene is a monoterpenoid with various toxic activities including neurotoxic effects and inhibition of reproduction and growth regulatory effects in several species of insects.

## 5. Conclusions

Based on our results, it can be concluded that chilies, garlic and, to a lesser extent peppermint, might serve as alternatives to insecticides on rural farms in tropical and subtropical regions. Insecticides are costly and not sustainable in the long run due to environmental contamination [17]; the use of these plant products would be cost effective and sustainable, especially considering that these plants are easy to grow. The plant products tested contain a range of bioactive chemicals, many of which are selective and have little or no harmful effect on non-target organisms and the environment [18]. In addition, these products are safe to users as evidenced by the fact that they are used as culinary spices and herbs.
